# Relationship between the triglyceride–glucose index and coronary artery calcification in asymptomatic, non-diabetic patients undergoing maintenance hemodialysis

**DOI:** 10.1080/0886022X.2023.2200849

**Published:** 2023-05-03

**Authors:** Hong Ding, Jinhua Zhu, Ying Tian, Li Xu, Lei Song, Ying Shi, Dongxing Mu, Ruoxin Chen, Hong Liu, Bicheng Liu

**Affiliations:** a Institute of Nephrology, People’s Hospital of Yangzhong city, Zhenjiang, Jiangsu Province, China; b Institute of Nephrology, Zhongda Hospital, Southeast University School of Medicine, Nanjing, Jiangsu Province, China

**Keywords:** Triglyceride–glucose index, cardiovascular disease, coronary artery calcification, non-diabetic, maintenance hemodialysis

## Abstract

**Objective:**

Coronary artery calcification (CAC) is positively and independently associated with cardiovascular disease (CVD) in patients undergoing maintenance hemodialysis (MHD). Insulin resistance is independently associated with CAC and is an important risk factor for CVD. The triglyceride–glucose (TyG) index is a reliable biomarker of insulin resistance. This cross-sectional, observational study aimed to investigate the relationship between the TyG index and CAC in asymptomatic non-diabetic patients undergoing MHD.

**Methods:**

The quantitative coronary artery calcification score (CACS) was calculated and expressed using the Agatston score. The TyG index was calculated as ln [fasting triglyceride (mg/dL) × fasting glucose (mg/dL)/2]. Multiple Poisson regression analysis, Spearman correlation analysis, and receiver operating characteristic (ROC) curves were used to investigate the relationship between the TyG index and CAC.

**Results:**

The 151 patients were divided into three groups according to the tertiles of the TyG index. With an increase in the TyG index, the CACS significantly increased (Spearman’s rho = 0.414, *p* < 0.001). Poisson regression analysis indicated that the TyG index was independently related to the presence of CAC (prevalence ratio, 1.281 [95% confidence interval, 1.121–1.465], *p* < 0.001). Furthermore, ROC curve analysis showed that the TyG index was of value in predicting the CAC in asymptomatic non-diabetic patients undergoing MHD, with an area under the curve of 0.667 (*p* = 0.010).

**Conclusion:**

The TyG index is independently related to the presence of CAC in asymptomatic, non-diabetic patients undergoing MHD.

## Introduction

Cardiovascular disease (CVD) is the leading cause of death among patients undergoing maintenance hemodialysis (MHD), and its associated relative risk of death is reported to be 20 times higher than that in the general population [1,2]. This high cardiovascular mortality is closely associated with vascular calcification [3], which frequently occurs and progresses almost universally in patients with end-stage renal disease [4,5]. Coronary artery calcification (CAC) is independently and significantly associated with the risk of developing CVD, myocardial infarction, and heart failure in patients undergoing MHD [6].

CAC is an early sign of coronary atherosclerosis, which is the basic lesion in coronary artery disease [7]. CAC is an important aspect of coronary artery disease [8]. Regardless of the risk factors or symptoms present in patients, CAC develops with the progression of atherosclerosis and is a predictor of the overall disease burden [9]. CAC, a sensitive marker used to detect the existence of early coronary artery atherosclerosis, can be determined using multidetector computed tomography (CT) [10,11]. However, the downside of CT as a screening test is that it is associated with a risk of radiation exposure. Therefore, it is of great clinical significance to search for simple and reliable factors that may reflect the presence of CAC.

Insulin resistance is closely associated with adverse outcomes in CVD [12,13] and has been demonstrated to be independently associated with CAC [14]. In addition, insulin resistance is associated with an increased risk of hyperglycemia and dyslipidemia, which in turn increase the risk of inflammation, coagulation abnormalities, and atherosclerosis and can promote the formation of vascular calcification [15,16]. The triglyceride–glucose (TyG) index, a reliable surrogate marker for insulin resistance [17,18], is a substantial risk factor for the development of CAC [19]. Previous studies have identified a strong relationship between the TyG index and CAC in different clinical conditions [20,21].

However, as data on the association between the TyG index and CAC in asymptomatic patients undergoing MHD are limited, we aimed to evaluate the association between them in the hopes of providing an economical and convenient index for the clinical evaluation of CAC in such patients.

## Materials and methods

### Study design and participants

**Figure 1.**
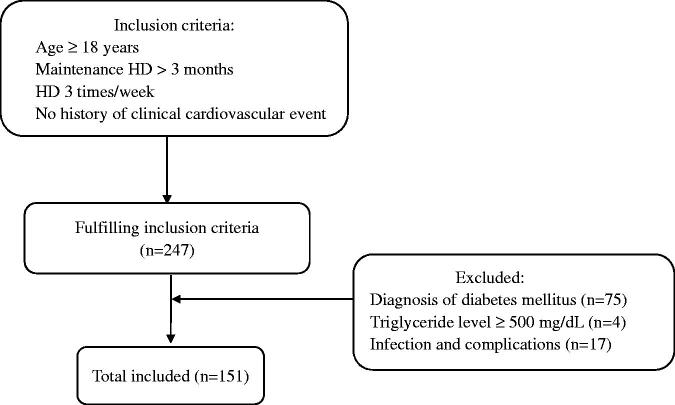
Enrollment flowchart for this study. HD: hemodialysis.

This was a cross-sectional, observational study. Patients who were undergoing regular hemodialysis at People’s Hospital of Yangzhong city were selected as study participants (Figure 1). The inclusion criteria were as follows: (a) age ≥18 years; (b) stable hemodialysis for >3 months; (c) regular hemodialysis 3 times/week for 4 h each time; (d) no history of CAD, cardiac pacemaker or defibrillator implantation, or suspected symptoms, such as chest pain and heart palpitations; and (e) agreement to undergo chest CT examination. The exclusion criteria were as follows: (a) diagnosis of diabetes mellitus by a doctor; (b) elevated triglyceride levels (≥ 500 mg/dL); (c) presence of malignant tumors, acute severe infection, connective tissue disease, severe metabolic diseases, decompensated chronic liver disease, hematologic diseases, or use of hormones in the past 3 months; (d) presence of severe cognitive impairment or mental illness; (e) pregnancy or lactation; and (f) lack of complete data.

The study complied with the principles of the Declaration of Helsinki and was approved by the ethics committee of the People’s Hospital of Yangzhong city (batch number: 2022053). Informed consent was obtained from all patients.

### Clinical data

Information on patient demographics (age and sex) and clinical characteristics (history of smoking, alcohol consumption, hypertension, diabetes, phosphorus binder therapy, CVD, and dialysis duration) was systematically collected. Individuals who smoked regularly in the past 6 months were considered current smokers, and those who consumed alcohol more than 3 times per week were considered current drinkers. Body mass index (BMI) was calculated by dividing the patient’s weight (kg) by their height squared (m^2^). Blood samples were collected in the morning after fasting overnight. Clinical data (blood glucose, triglycerides, cholesterol, high-density lipoprotein cholesterol [HDL-C], low-density lipoprotein cholesterol [LDL-C], hemoglobin, albumin, calcium, phosphorus, 25(OH)D_3_, alkaline phosphatase, parathyroid hormone [PTH], C-reactive protein [CRP], ferritin, uric acid [UA], and fractional clearance index for urea [Kt/V]) of the preceding 3 months were retrieved from electronic medical records. Two clinical medical staff members checked the patients’ electronic medical records for relevant data. The TyG index was calculated using ln (triglycerides [mg/dL] × glucose [mg/dL]/2). All participants were categorized into three groups based on the tertiles of the TyG index.

### Calcium score calculation (Agatston method)

CAC was quantified using chest CT. CT acquisition was performed using a 256-detector row CT scanner (Revolution CT; GE Healthcare, Milwaukee, WI, USA). A dedicated, prospective ECG-triggered calcium score scan at 75% (HR <65 beats/min) or 45% (HR ≥ 65 beats/min) of the cardiac cycle was performed. The degree of CAC was quantified using non-enhanced scanning. Data were uploaded to an advanced workstation (AW4.7; GE Healthcare) for analysis. CT images were analyzed by two experienced radiologists, who manually mapped the lesion areas at each level using a special customized calculation software (smart score 4.0; GE Healthcare) through which the Agatston scores were obtained for each patient.

According to the coronary artery calcification score (CACS), patients were divided into the non-calcification group (0 score) and calcification group (>0 score). The calcification group was further divided into mild- (1 ∼ 99 score), moderate- (100 ∼ 399 score) and severe- groups (≥400 score) [22].

### Statistical analyses

Continuous variables with a normal distribution were expressed as the mean ± standard deviation and compared using one-way analysis of variance tests. Non-normally distributed continuous variables were expressed using medians and quartiles, and the Kruskal–Wallis test was used for comparison between groups. For categorical variables, chi-square tests were used to analyze differences among the three groups. The relationship between CAC and various clinical parameters was analyzed using Spearman’s correlation, with the CACS as a continuous numerical variable. Multiple Poisson regression analysis with robust variance was performed with those variables showing a *p* < 0.20 in the bivariate analysis. The area under the curve (AUC) of the receiver operating characteristic (ROC) curve was used to calculate the predictive power of the TyG index for CAC. The best cutoff value was calculated using the Youden index [23]. All statistical analyses were performed using the SPSS statistical package (version 26.0; SPSS, Inc., Chicago, IL, USA). For figures, Prism version 9.0 (GraphPad Software, La Jolla, California, USA) was used. The significance level was set at *p* < 0.05.

## Results

### Baseline characteristics

A total of 151 asymptomatic, non-diabetic patients undergoing MHD were enrolled, among whom 84 (55.63%) were male and 67 (44.37%) were female. Table 1 presents the clinical characteristics of the participants according to tertiles of the TyG index. The mean age of the participants at baseline was 56.66 ± 12.43 years, and the mean duration of dialysis was 49.00 (21.00–99.00) months. BMI, diastolic blood pressure (DBP), fasting blood glucose, triglyceride, HDL-C, LDL-C, phosphorus, CRP, and PTH levels were significantly different among the groups. No significant differences were found in age, systolic blood pressure (SBP), hypertension, smoking, alcohol consumption, phosphorus binder therapy, total cholesterol, hemoglobin, albumin, alkaline phosphatase, 25(OH)D_3_, calcium, UA, ferritin, and Kt/v. Importantly, the presence of CAC and the CACS significantly increased with an increase in the TyG index.

**Table 1.** Clinical characteristics of the participants according to TyG index tertiles.

	Total	TyG index			*p* Value
		Tertile 1 (7.32–8.33)	Tertile 2 (8.34–8.81)	Tertile 3 (8.82–10.32)	
Number of participants, (n)	151	50	50	51	–
Sex (male/female), (n)	84/67	30/20	25/25	29/22	0.589
Age, (y)	56.66 ± 12.43	56.80 ± 12.62	56.38 ± 12.82	56.78 ± 12.10	0.982
BMI, (kg/m^2^)	22.70 ± 3.75	22.13 ± 3.32	22.04 ± 3.02	23.91 ± 4.49	0.017
Smoking, *n* (%)	30 (19.87%)	7 (14.00%)	11 (22.00%)	12 (23.53%)	0.437
Alcohol consumption, *n* (%)	28 (18.54%)	10 (20.00%)	11 (22.00%)	7 (13.73%)	0.535
Hypertension, *n* (%)	120 (79.47%)	41 (82.00%)	43 (86.00%)	36 (70.59%)	0.137
SBP, (mmHg)	144.77 ± 23.15	149.76 ± 21.67	139.24 ± 22.49	145.31 ± 24.41	0.073
DBP, (mmHg)	80.70 ± 11.48	84.48 ± 10.63	77.54 ± 10.07	80.08 ± 12.69	0.009
Fasting plasma glucose, (mmol/L)	4.70 (4.20–5.10)	4.55 (4.00–4.83)	4.55 (4.20–5.10)	4.90 (4.30–5.90)	0.004
Triglyceride, (mmol/L)	1.39 (0.95–2.13)	0.82 (0.62–1.03)	1.39 (1.24–1.58)	2.41 (2.12–3.08)	<0.001
TyG index	8.55 (8.17–9.03)	8.04 (7.72–8.18)	8.53 (8.42–8.70)	9.14 (9.02–9.49)	<0.001
Total cholesterol, (mmol/L)	4.33 ± 1.04	4.08 ± 0.92	4.50 ± 1.11	4.40 ± 1.05	0.110
HDL-C, (mmol/L)	1.09 (0.91–1.32)	1.26 (0.98–1.44)	1.09 (0.87–1.31)	1.00 (0.84–1.10)	<0.001
LDL-C, (mmol/L)	2.13 ± 0.74	1.89 ± 0.60	2.28 ± 0.77	2.20 ± 0.78	0.021
Hemoglobin, (g/L)	104.15 ± 18.31	103.64 ± 15.93	103.80 ± 19.19	104.98 ± 19.87	0.923
Albumin, (g/L)	41.90 (39.60–44.60)	41.90 (39.10–44.93)	41.75 (39.75–44.13)	42.10 (39.60–44.60)	0.852
Alkaline phosphatase, (U/L)	73.20 (57.00–94.00)	66.50 (54.75–81.00)	76.50 (59.50–103.00)	78.00 (60.00–98.00)	0.068
25(OH)D_3_, (ng/ml)	29.31 ± 11.65	30.47 ± 11.48	28.81 ± 12.31	28.67 ± 11.29	0.693
Calcium, (mmol/L)	2.18 (2.08–2.31)	2.17 (2.08–2.27)	2.17 (2.03–2.30)	2.26 (2.09–2.38)	0.174
Phosphorus, (mmol/L)	1.98 ± 0.58	1.84 ± 0.51	1.91 ± 0.53	2.17 ± 0.65	0.012
UA, (μmol/L)	421.00 (372.00–484.00)	407.50 (366.75–447.50)	415.00 (372.25–490.50)	443.00 (401.00–502.00)	0.071
CRP, (mg/L)	0.81 (0.50–5.08)	0.50 (0.50–1.28)	1.03 (0.50–7.63)	2.52 (0.50–7.06)	<0.001
Ferritin, (ng/ml)	97.69 (48.90–231.65)	141.49 (46.84–272.49)	85.49 (48.68–220.07)	95.70 (47.45–146.38)	0.399
PTH, (pg/mL)	221.40 (108.40–392.00)	203.25 (79.03–333.23)	168.65 (99.33–377.55)	336.00 (166.50–445.80)	0.013
Phosphorus binder therapy, *n* (%)	14 (9.27%)	3 (6.00%)	3 (6.00%)	8 (15.69%)	0.152
Duration of dialysis, (month)	49.00 (21.00–99.00)	42.00 (22.00–77.25)	41.00 (17.75–78.75)	67.00 (35.00–121.00)	0.032
Kt/V	1.26 ± 0.04	1.27 ± 0.04	1.26 ± 0.03	1.26 ± 0.03	0.087
Presence of CAC, *n* (%)	127 (84.11%)	36 (72.00%)	44 (88.00%)	47 (92.16%)	0.016
CACS	302.20 (29.60–976.32)	66.53 (0–469.18)	247.76 (37.35–827.61)	666.58 (165.10–2523.46)	<0.001

BMI: body mass index; SBP: systolic blood pressure; DBP: diastolic blood pressure; HDL-C: high-density lipoprotein cholesterol; LDL-C: low-density lipoprotein cholesterol; UA: uric acid; CRP: C-reactive protein; PTH: parathyroid hormone; Kt/v: fractional clearance index for urea; CAC: coronary artery calcification; CACS: coronary artery calcification score.

### Association between CAC and TyG index

Spearman’s correlation analysis was performed to examine the relationship between CAC and various clinical parameters (Table 2). The CACS was used as the dependent variable, and indicators that may affect CAC were used as independent variables. The results showed that age, duration of dialysis, HDL-C, albumin, and CRP were related to CAC. Remarkably, CAC was significantly correlated with the TyG index (Spearman’s rho = 0.414; *p* < 0.001). As shown in Table 3, multiple regression analysis showed that the TyG index was positively associated with CAC (prevalence ratio, 1.281; [95% confidence interval, 1.121–1.465]; *p* < 0.001).

**Table 2.** Correlations between the CACS and risk factors.

	r	*p* Value
Sex	−0.105	0.200
Age	0.282	**<0.001**
BMI	0.051	0.531
Duration of dialysis	0.410	**<0.001**
Hemoglobin	−0.119	0.146
Albumin	−0.277	**0.001**
TyG index	0.414	**<0.001**
HDL-C	−0.175	**0.032**
Alkaline phosphatase	0.092	0.262
25(OH)D_3_	−0.106	0.193
Calcium	0.038	0.640
Phosphorus	0.007	0.936
UA	−0.010	0.907
CRP	0.372	**<0.001**
Ferritin	0.059	0.469
PTH	0.106	0.196

BMI: body mass index; HDL-C: high-density lipoprotein cholesterol; LDL-C: low-density lipoprotein cholesterol; UA: uric acid; CRP: C-reactive protein; PTH: parathyroid hormone; CACS: coronary artery calcification score.

Bold values are statistically significant at *p* < 0.05.

**Table 3.** Poisson regression analysis of CAC with the TyG index.

Variables	Crude PR (CI 95%)	P-value	Adjusted PR (CI 95%)	*p* Value
**Age**				
≥60	2.732(2.499–2.986)	<0.001	1.136(1.031–1.253)	0.010
<60	–	–	–	–
**Sex**				
male	1.046(0.923–1.184)	0.483		
female	–	–		
**BMI**				
≥25	1.021(0.886–1.177)	0.772		
<25	–	–		
**Smoking**				
yes	1.048(0.914–1.202)	0.499		
no	–	–		
**Alcohol consumption**				
yes	1.049(0.907–1.215)	0.518		
on	–	–		
**Hypertension**				
yes	1.081(0.907–1.289)	0.384		
no	–	–		
**Phosphorus binder therapy**				
yes	1.098(0.901–1.338)	0.353		
on	–	–		
**TyG index**				
tertile 1	–	–	–	–
tertile 2	1.230(1.042–1.451)	0.015	1.164(1.015–1.333)	0.029
tertile 3	1.398(1.199–1.630)	<0.001	1.281(1.121–1.465)	<0.001
**Dialysis duration**	2.453(2.224–2.706)	<0.001	1.002(1.001–1.003)	<0.001
**Calcium**	0.990(0.989–0.991)	<0.001	0.993(0.992–0.995)	<0.001
**Phosphorus**	1.010(0.911–1.119)	0.853		
**HDL-C**	0.813(0.664–0.996)	0.046	1.010(0.849–1.201)	0.914
**Albumin**	0.973(0.961–0.985)	<0.001	0.983(0.969–0.997)	0.020
**UA**	1.000(0.999–1.001)	0.701		
**CRP**	1.018(1.012–1.025)	<0.001	1.010(1.004–1.016)	0.002

PR: prevalence ratio; CI: confidence interval; BMI: body mass index; HDL-C: high-density lipoprotein cholesterol; UA: uric acid; CRP: C-reactive protein.

**Figure 2.**
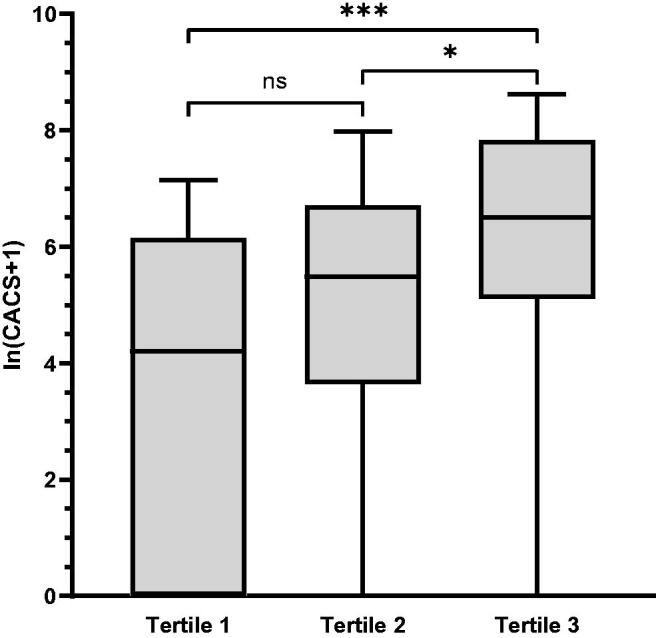
The CACS was grouped according to the TyG index tertiles. CACS: coronary artery calcification score. *<0.05; **<0.01; ***<0.001.

As shown in Figure 2, there were significant differences in the CACS among the three groups according to the TyG index tertiles (*p* < 0.001). The CACS was significantly different between the tertile 3 group and the tertile 1 and tertile 2 groups (*p* < 0.001 and *p* = 0.029, respectively). In addition, with an increase in the TyG index, the median CACS gradually increased (Figure 2).

**Figure 3.**
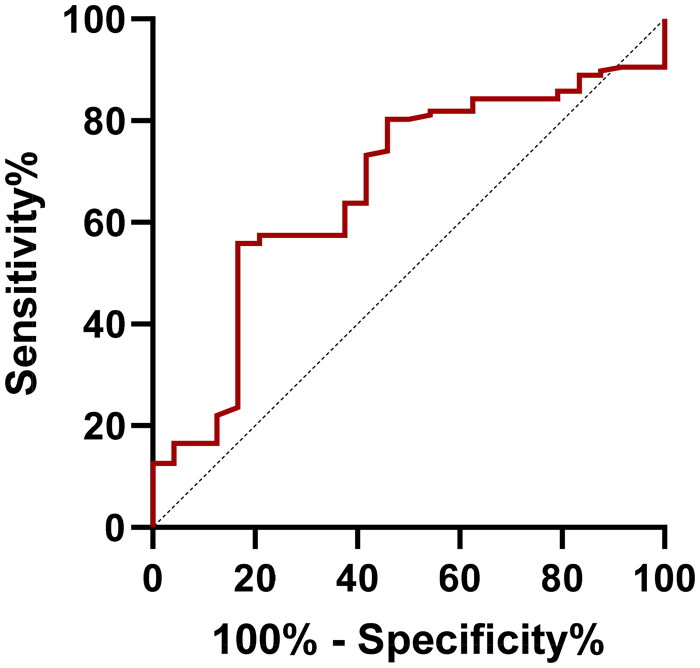
ROC curves of the TyG index (AUC = 0.667). ROC: receiver operating characteristic; AUC: area under the curve.

According to ROC curve analysis, the AUC for the TyG index was 0.667 (*p* = 0.010; Figure 3). The cutoff value of the TyG index was 8.55, with a sensitivity of 55.90% and a specificity of 83.30%.

## Discussion

In the present study, we observed a significant association between the TyG index and CAC in asymptomatic, non-diabetic patients undergoing MHD. After adjusting for confounding factors, an independent and positive association was observed between the TyG index and CAC in asymptomatic non-diabetic patients undergoing MHD.

CAC can be present in a wide range of cardiovascular conditions, from early asymptomatic coronary artery disease to severe CVD [24]. It is an important indicator of subclinical atherosclerosis [25,26] and is associated with major cardiovascular events and mortality [6]. Studies have demonstrated that disturbances in phosphorus and calcium metabolism, high levels of PTH, and inflammatory factors can stimulate vascular wall smooth muscle cells, induce osteoblast-like function, secrete bone morphogenetic proteins, and accelerate vascular calcification [27–29]. The results of a cohort study with 7 years of follow-up showed a higher mortality rate in dialysis patients with severe CAC [30]. Therefore, it is of great clinical significance to explore the relationship between the TyG index and CAC in asymptomatic non-diabetic patients undergoing MHD.

As a surrogate marker of insulin resistance, the TyG index is closely related to high-risk factors for CVD as well as adverse cardiovascular prognosis [19]. Insulin resistance can promote vascular calcification not only through mechanisms involving systemic factors, such as calcium and phosphorus metabolism disorders and pro-inflammatory states [16,31], but also at the cellular level [14,32]. Insulin resistance leads to impaired glucose metabolism and accelerated vascular calcification by blocking signaling pathways mediated by insulin receptors on the cell membranes of vascular smooth muscle cells, endothelial cells, and macrophages [14,33]. In addition, our previous study showed that sarcopenia is a common complication and is closely related to insulin resistance in non-diabetic patients undergoing MHD [34]. Sarcopenia can cause decreased expression of myokines, such as irisin, and low irisin is an independent risk factor for vascular calcification [35,36]. This may be related to the high incidence of vascular calcification in patients undergoing MHD, which warrants further investigation.

In recent years, increasing research has demonstrated that the TyG index is independently correlated with CAC [20,37,38]. In 2019, Park et al. observed that even in the absence of traditional CVD risk factors, there was an independent association between the TyG index and CAC in Korean adults [20]. Following this, in 2020, results from a large cohort study conducted by Won et al. showed that a high TyG index was strongly associated with an increased risk of CAC progression in asymptomatic adults [39]. Consistent with previous studies, our study showed that the TyG index was closely related to CAC in non-diabetic patients undergoing MHD.

Over the past decade, numerous studies have greatly improved our understanding of the mechanisms underlying vascular calcification in patients undergoing MHD; however, many questions remain to be answered. Given the impact of CAC on adverse clinical outcomes, particularly in patients undergoing MHD, the identification of reliable and simple independent predictors of CAC in clinical practice is a significant concern. CAC can be detected using CT [40]. However, the disadvantage of this method as a screening test is its associated risk of radiation exposure. The TyG index is a simple and affordable marker; therefore, we sincerely hope that it can play a role in early identification of CAC so that timely interventions can be implemented to improve the prognosis of the disease.

Despite the efforts made in this study, there were some limitations that should be mentioned. First, owing to the limitations of the method used to measure CAC, we were unable to distinguish between intimal and medial calcifications. Second, due to the presence of unavoidable clinical confounding factors, the AUC for the TyG index was only 0.667 (*p* = 0.010). Third, the prevalence rate in the group with low TyG value in Table 1 was higher, but the CACS were significantly lower than those in the other two groups. Therefore, a comprehensive assessment should be made combining prevalence and CACS. Fourth, our study population was relatively small, and the research participants were all from a single center. Therefore, multicenter studies with larger sample sizes should be considered in the future. Fifth, this was a clinical cross-sectional observational study, and we were unable to definitively establish causality. Thus, the precise causal relationship between the TyG index and CAC remains controversial.

## Conclusions

The TyG index was correlated with CAC in asymptomatic non-diabetic patients on MHD. However, these findings should be interpreted with caution, as further studies are needed to elucidate the precise relationship between the TyG index and CAC.

## Data Availability

The data analyzed in this study are available from the corresponding author upon reasonable request.
